# Kinetic coupling in distal foot joints during walking

**DOI:** 10.1186/s13047-023-00643-x

**Published:** 2023-07-25

**Authors:** Lauren R. Williams, Elisa S. Arch, Dustin A. Bruening

**Affiliations:** 1 Brigham Young University, Provo, UT 84602 USA; 2 University of Delaware, Newark, DE 19716 USA

**Keywords:** Medial longitudinal arch, Metatarsophalangeal joint, Multisegment foot, Foot energetics, Walking

## Abstract

**Background:**

Kinematic coupling between the first metatarsophalangeal (MTP) and midtarsal joints is evident during gait and other movement tasks, however kinetic foot coupling during walking has not been examined. Furthermore, contributing factors to foot coupling are still unclear. Therefore, the purpose of this study was to investigate kinematic and kinetic coupling within the foot by restricting MTP motion during overground walking. We hypothesized that when the MTP joint was prevented from fully extending, the midtarsal joint would achieve less peak motion and generate less positive work compared to walking with normal MTP motion.

**Methods:**

Twenty-six individuals participated in this randomized cross-over study. Using motion capture to track motion, participants walked at 1.3 m/s while wearing a brace that restricted MTP motion in a neutral (BR_NT) or extended (BR_EX) position. Additionally, participants walked while wearing the brace in a freely moveable setting (BR_UN) and with no brace (CON). A pressure/shear sensing device was used to capture forces under each foot segment. During stance, peak joint motion and work were calculated for the MTP and midtarsal joints using inverse dynamics. A series of ANOVAs and Holm post hoc tests were performed for all metrics (alpha = 0.05).

**Results:**

The brace successfully decreased peak MTP motion by 19% compared to BR_UN and CON. This was coupled with 9.8% less midtarsal motion. Kinetically, the work absorbed by the MTP joint (26–51%) and generated by the midtarsal joint (30–38%) were both less in BR_EX and BR_NT compared to BR_UN.

**Conclusion:**

Implications and sources of coupling between the MTP and midtarsal joints are discussed within the context of center of pressure shifts and changes to segmental foot forces. Our results suggest that interventions aimed at modulating MTP negative work (such as footwear or assistive device design) should not ignore the midtarsal joint.

**Supplementary Information:**

The online version contains supplementary material available at 10.1186/s13047-023-00643-x.

## Background

Intersegmental coordination within the foot is apparent in activities ranging from simple passive movement [1–3], to walking [2, 4], and running [5–7]. Across all these activities, it is evident that as the metatarsophalangeal (MTP) joints are extended, the medial longitudinal arch (MLA) rises concomitantly (i.e., midtarsal plantarflexion). In addition to this observed kinematic coupling between the MTP and midtarsal joints, kinetic coupling may also be present, as energy absorption at the MTP joint occurs jointly with midtarsal energy generation [3–8]. Furthermore, alterations in MTP motion or power due to task manipulation (e.g., changing walking speed [9] or varying running foot strike pattern [5]) show proportional changes at the midtarsal joint, further reinforcing that these two joints are functionally linked. While past studies support functional coupling within the foot, the extent of this coupling and the relative contributions to this coupling remains unclear.

While it is difficult to fully isolate the various factors contributing to foot coupling, systematically manipulating foot joints could further our understanding of foot mechanics. Previous studies manipulating MTP mechanics have been useful, but there are still gaps within this research. Kinetic coupling during heel raises [3] and kinematic coupling during walking [2] have been reported; however, kinetic coupling within the foot during walking and the contributing factors to this coupling have not yet been explored. Further understanding of foot coupling could provide insight for interventions aimed at modulating MTP negative work (such as footwear or assistive device design).

The purpose of this study was to probe the extent of coupling within the foot by systematically manipulating motion at the first MTP joint during overground walking. Specifically, we used a brace to restrict extension at the first MTP joint (henceforth referred to as MTP). We hypothesized that when the MTP joint was prevented from fully extending, the midtarsal joint would have less range of motion (ROM) compared to walking with normal MTP motion. Secondly, we hypothesized that negative MTP work and positive midtarsal work would both decrease when the MTP joint was prevented from fully extending compared to walking with normal MTP motion. In addition, we investigated accompanying changes to center of pressure (COP), joint moment, and segmental force, anticipating that these would provide some insights into the mechanisms and contributing factors important to this coupling.

## Methods

### Participants

Twenty-six individuals (15 males, 11 females; age: 24.65 ± 4.37; height (m): 1.74 ± 0.09; weight (kg): 71.2 ± 10.72) volunteered to participate in this randomized cross-over study. Participants were excluded if they had any pathologies or injury history that might affect walking. Before any data collection, participants were asked to thoroughly read and sign an IRB-approved informed consent form (Protocol # X2019-383).

### Procedures

First, participants’ height and weight were measured. Then, the left foot was outfitted with 15 markers according to a multi-segment foot marker set [3] while the right foot was outfitted with a simple, single segment foot model to track gait cycles.

**Fig. 1:**
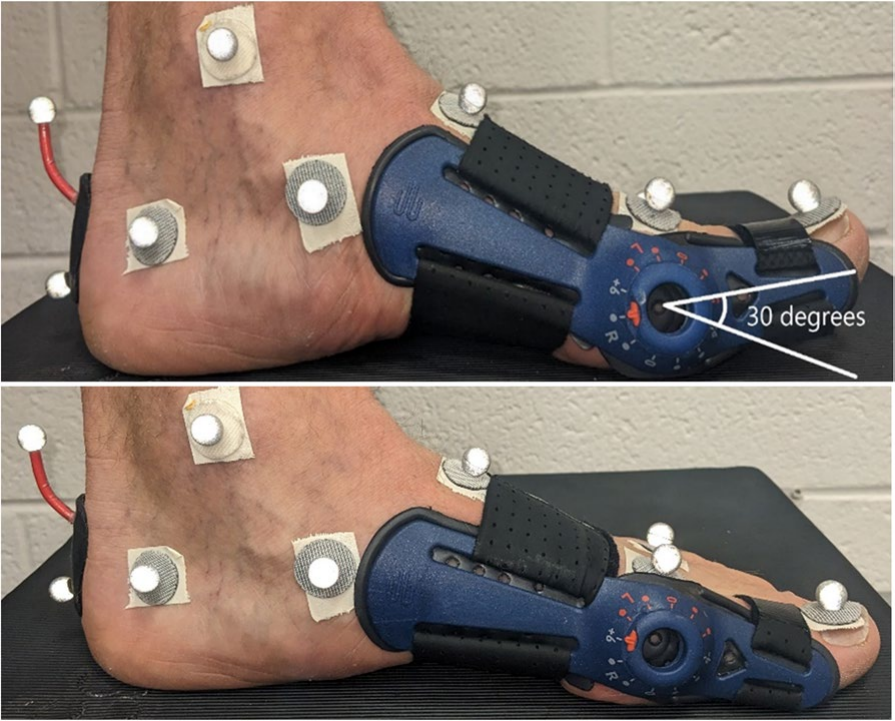
A subject wearing the brace positioned in 30 degrees of MTP extension (BR_EX; top image) and brace locked in zero degrees of extension (BR_NT; bottom image)

After markers were placed, subjects walked along a 5.5 m walkway at a controlled walking speed (1.3 m/s) for four order-randomized conditions. The speed of each subject was monitored with timing lights (Brower, Salt Lake City, UT, USA) and each trial had to be within 0.1 m/s of the target speed in order to be considered successful. A baseline condition was performed with subjects walking barefoot (without the brace) to evaluate effects of wearing the brace (CON). Brace conditions included subjects walking while wearing the brace on the left foot (Fig. 1; ValguLoc II, Bauerfeind, Inc. Atlanta, GA, USA), when it was unlocked so that the MTP joint moved freely (BR_UN). Additionally, subjects walked with MTP motion restricted in two different ways, either locked at zero degrees of extension (BR_NT) or locked into 30 degrees of extension (BR_EX). This brace was chosen as it can limit MTP extension without impeding marker placement. The starting position on the walkway was adjusted by researchers to prevent targeted foot placement. At least three trials with accurate speed and successful foot placement on a commercial pressure/shear sensing device (FootSTEPS, ISSI, Dayton, OH, USA) were collected for the four conditions.

Motion data were collected with a 12-camera motion capture system (Qualisys, Gottberg, Sweden) at 100 Hz. Force, center of pressure, and free moment data for each foot segment were calculated using pressure and shear data from the FootSTEPS device, which was placed in the center of the walkway. This sensor consists of a stress-sensitive film, a camera (sampled at 50 Hz), and a force plate (sampled at 1000 Hz). During contact, film displacements are optically measured by the camera, then converted into vertical pressure and mediolateral and anteroposterior shear stress distributions using a finite element analysis. Further details about this device’s hardware and measurement validity are available by Goss et al. [10].

### Data analysis

Data analysis consisted of segment force identification, performed in custom LabView code (National Instruments, Austin, TX, USA), followed by model kinematics and inverse dynamics, performed in Visual 3D software (C-Motion, Inc. Germantown, MD, USA).

For segment force identification, pressure and shear data from the FootSTEPS device were first calibrated to the force platform and up-sampled to 100 Hz. Next, synchronized marker positions were projected onto a composite pressure footprint in order to identify the boundaries of each segment (hindfoot, forefoot, and hallux), ensuring that they matched the model endpoints [3]. Boundary identification was automated using a combined geometric and anatomical masking algorithm. This involved first manually creating a template from one representative footprint. Template boundary line points were expressed as barycentric coordinates relative to marker triads and then applied to all other footprints using these marker positions (i.e. anatomical mask). The boundaries between the forefoot and hallux segments were then adjusted using a gradient descent optimization (i.e. geometric mask). After segment boundary identification, full segment ground reaction forces (GRFs, consisting of vertical and horizontal forces, centers of pressure, and free moments) were constructed from the segmental pressure and shear distributions, summing across segment area. Additional details regarding these steps are contained in [11].

The segmental GRFs were imported into Visual 3D along with the motion capture trajectories. A previously detailed multi-segment foot model was applied [3], and forces were applied to each segment. Briefly, the model contains anatomically aligned hindfoot, forefoot, and hallux segments separated by landmark-defined midtarsal and MTP joints. The midtarsal joint was defined as the midpoint between markers on the navicular and cuboid joints, while the MTP joint was projected to the center of the 1^st^ metatarsal head. Foot and lower extremity joint angles were calculated from adjacent segment reference frames using a typical Euler/Cardan rotation sequence (1-flexion/extension, 2-abduction/adduction, 3-internal/external rotation). Internal joint moments and joint power were calculated using inverse dynamics, expressing moments in the proximal segment reference frame.

Time-normalized angle, moment, and power waveforms for the ankle, midtarsal, and MTP joints were created for visualization. The knee and hip angle waveforms were also created and visually analyzed to rule out other compensations. Range of motion (ROM) was calculated during stance for the midtarsal and MTP joints from peak plantarflexion to peak dorsiflexion angle. Joint work was calculated as the integral of the power curve during stance phase. For ankle negative work, integration started at 20% of stance phase to exclude the small positive work evident during early stance. The location of hallux COP was calculated by taking the weighted average in the anterior/posterior direction. Then, the hallux COP location was normalized to segment length prior to calculating means across subjects. Peak plantarflexion moments for the MTP and midtarsal joints were identified from the moment curves during stance phase. Similarly, the peak vertical GRF for the forefoot and hallux segments were identified during stance phase. And lastly, all metrics were averaged across the three trials so that one value per condition could be analyzed for statistics.

### Statistics

A series of within-subjects repeated measures ANOVAs were performed for all metrics. Primary variables consisted of MTP and midtarsal peak angles and ROM, negative MTP work and positive midtarsal work. Secondary variables were hallux COP, peak MTP and midtarsal plantarflexion joint moments, and peak forefoot and hallux vertical GRF. For each ANOVA, Mauchly’s test for sphericity was tested and corrected for if necessary. A Holm post hoc test was applied if the main effect showed significance (α = 0.05).

## Results

### Overview

**Fig. 2:**
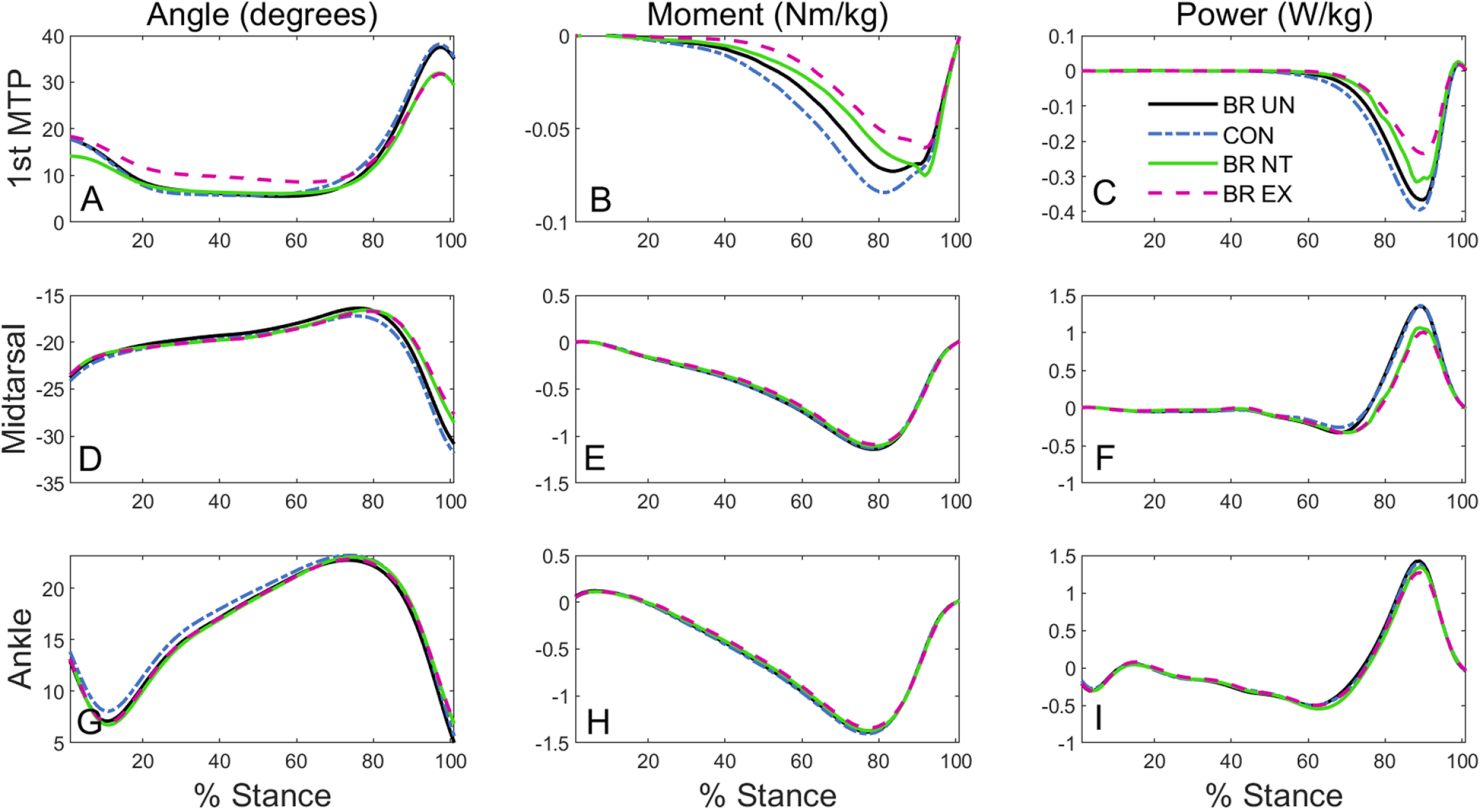
Joint angles, moments, and power profiles during stance phase. For angle and moment profiles, dorsiflexion is positive and plantarflexion is negative

The toe brace did not fully lock the MTP joint during BR_EX and BR_NT, but it did restrict motion and create an effective contrast between conditions (Fig. 2A). For both locked conditions (BR_NT and BR_EX), peak MTP extension was similar, approximately seven degrees (19%) reduced compared with the unconstrained conditions (CON and BR_UN). Through midstance, BR_EX maintained some toe extension, with mean extension approximately three degrees higher than all other conditions, thus overall ROM at the MTP in BR_EX was a few degrees less than BR_NT (4 degrees). The presence of the brace by itself did have some minor effects, as noted in specific comparisons between unconstrained conditions below. Descriptively, we focused on comparisons between locked and unlocked brace conditions as our primary focus, with differences between unconstrained conditions noted secondarily. We also visually analyzed the knee and hip angles for unintended compensations without finding any apparent differences among conditions. These plots are available as additional files (Additional file 1.pdf). A few differences were noted in ankle mechanics, and these are included in the main results.

### Kinematics

Kinematic coupling was apparent in the distal foot as the midtarsal joint underwent less motion during walking when MTP joint motion was restricted (Fig. 2 A and D), with ROM and peak metrics at both joints showing significant main effects (Table 1). Peak midtarsal plantarflexion was approximately three degrees (~ 9.8%) less for the two locked brace conditions compared to BR_UN. Similarly, midtarsal ROM was approximately three degrees (~ 20%) less in BR_NT and BR_EX. There were no statistical differences in the ROM or peak angles for the ankle joint. When comparing BR_UN to CON, there were some small differences within the peak angles for the MTP and midtarsal joints. Wearing the brace resulted in less peak foot joint motion: 1.4 degrees less for the MTP joint (4%) and 1.3 degrees less for the midtarsal joint (3.5%).

**Table 1:** Joint metrics during stance for each condition (mean ± SD)

	***CON***	***BR_UN***	***BR_NT***	***BR_EX***	***P-value***
***Primary Metrics***					
**Peak Joint Angle (deg)**					
Midtarsal Plantarflexion	-32.86 ± 4.91^bcd^	-31.54 ± 4.78^acd^	-29.34 ± 5.23^ab^	-28.43 ± 5.60^ab^	< 0.001*
1^st^ MTP Dorsiflexion	40.2 ± 5.5^bcd^	38.8 ± 5.2^acd^	32.9 ± 5.6^ab^	33.2 ± 5.5^ab^	< 0.001*
**Joint ROM (deg)**					
Midtarsal	16.28 ± 3.14^ cd^	15.61 ± 3.1^ cd^	13.04 ± 3.4^ab^	12.55 ± 3.7^ab^	< 0.001*
1^st^ MTP	35.63 ± 5.39^ cd^	35.37 ± 5.48^ cd^	27.42 ± 5.51^ab^	25.5 ± 5.81^ab^	< 0.001*
**Joint Work (J/kg)**					
Ankle Positive Work	0.122 ± 0.05	0.127 ± 0.05^ cd^	0.115 ± 0.05^b^	0.114 ± 0.05^b^	0.033*
Midtarsal Positive Work	0.117 ± 0.04^ cd^	0.117 ± 0.03^ cd^	0.086 ± 0.04^ab^	0.079 ± 0.03^ab^	< 0.001*
1^st^ MTP Negative Work	-0.045 ± 0.02^bcd^	-0.039 ± 0.01^acd^	-0.031 ± 0.01^abd^	-0.023 ± 0.01^abc^	< 0.001*
***Secondary Metrics***					
**Center of Pressure (cm)**					
Distance from 1^st^ MTP Joint Center^	3.34 ± 0.71^ cd^	3.48 ± 0.58	3.63 ± 0.63^a^	3.65 ± 0.62^a^	< 0.001*
**Peak Joint Moment (Nm/kg)**					
Midtarsal Plantarflexion	-1.17 ± 0.12^ cd^	-1.17 ± 0.11^ cd^	-1.12 ± 0.012^ab^	-1.12 ± 0.12^ab^	< 0.001*
1^st^ MTP Plantarflexion	-0.094 ± 0.02^d^	-0.086 ± 0.02	-0.09 ± 0.02^d^	-0.07 ± 0.03^ac^	0.003*
**Peak Segment Force (N/kg)**					
Forefoot Force	8.46 ± 0.84^bcd^	8.94 ± 0.86^acd^	9.39 ± 0.91^ab^	9.57 ± 0.94^ab^	< 0.001*
Hallux Force	2.85 ± 0.65^bcd^	2.58 ± 0.62^bd^	2.47 ± 00.66^ad^	2.14 ± 0.89^abc^	< 0.001*

Pairwise comparison results presented after Holm correction

^a^ Pairwise different from CON

^b^ Pairwise different from BR_UN

^c^ Pairwise different from BR_NT

^d^ Pairwise different from BR_EX

^ Weighted average and length-normalized for the hallux segment during stance

^*^ Significant main effect from ANOVA

### Kinetics

Similar to our kinematic findings, distal foot kinetic coupling was also apparent. The energy generated by the midtarsal joint and absorbed by the MTP joint was less in locked brace compared to unconstrained conditions (Fig. 2 C and F). Both the positive work at the midtarsal joint and the negative work at the MTP joint exhibited significant main effects (Table 1), with BR_EX and BR_NT performing less positive work at the midtarsal joint (30 and 38%, respectively) and less negative work at the MTP joint (26 and 51%, respectively) compared to BR_UN. Lastly, the ankle performed slightly more positive work (9.9%) during BR_UN compared to locked conditions. When comparing the two unconstrained conditions (BR_UN and CON), subjects performed slightly less (14%) negative work at the MTP joint while wearing the brace. There was no difference in the positive work at the midtarsal joint between CON and BR_UN.

There were significant main effects in hallux COP, peak joint moments, and peak segment forces. The hallux COP location for both locked brace conditions was approximately 0.3 cm farther forward on the hallux compared to CON and 0.15 cm farther than BR_UN (Fig. 4). The peak plantarflexion moment magnitude at the midtarsal joint decreased by 0.05 Nm/kg for both locked conditions. Additionally, the MTP peak moment magnitude decreased by 0.016 Nm/kg during BR_EX, but didn’t significantly change during BR_NT. The peak segmental forces increased at the forefoot and decreased at the hallux for the locked conditions. Specifically, peak forefoot force increased by 10.4% for BR_NT and 12.3% for BR_EX compared to CON while peak hallux force decreased by 14.29% for BR_NT and 28.5% for BR_EX. These measurements and results are further explored in the discussion. For peak forefoot force, there was a difference between CON and BR_UN. Additionally, for peak hallux force, BR_EX had less peak force compared to BR_NT while there was no statistical difference between the locked brace conditions for peak forefoot force.

## Discussion

The purpose of this study was to explore the extent of coupling within the foot and to investigate potential contributing factors through systematic manipulation of the MTP joint during walking. From previous work, we expected that when the MTP joint was locked (either in a neutral or extended position) the midtarsal joint would exhibit less ROM and less positive work generation concomitant with the decrease in MTP ROM and negative work absorption. Overall, the data collected in this study support our foot coupling hypotheses; when MTP mechanics are altered the midtarsal joint responds both kinematically and kinetically.

When the MTP joint was prevented from fully extending, the midtarsal joint was also proportionally affected, matching previous research showing kinematic coupling between these two foot joints. The decrease in ROM at the MTP joint induced by our locked brace conditions is comparable to other studies where MTP motion was altered through various mechanisms (e.g. hallux rigidus [12], wedge placed under the toes [13], and sandals with an upward curvature under the toes [14]). All these studies resulted in the MTP joint being more dorsiflexed throughout midstance and having less ROM in late stance compared to controls [12–14]. This was also observed in our locked brace conditions. Within our study, the reduction in MTP motion resulted in reduced motion at the midtarsal joint. This is consistent with previous research showing kinematic intersegmental coordination within the distal foot [2, 3], although two additional studies did not reach statistical significance [12, 13]. Stevens et al. showed a 2.9 degree mean difference in midtarsal ROM in hallux rigidus. This difference was similar in magnitude to the 3.0 degree (10 percent) difference we observed at the midtarsal joint for our locked brace conditions compared to normal walking. This similarity in magnitude is insightful considering that Stevens et al. results were in a clinical population while our results were due to direct manipulation of MTP mechanics. The lack of significance in Stevens et al. could simply be due to a lower subject number (*n* = 16 for hallux rigidus versus *n* = 26 for the current study), or to the total range of motion restricted at the MTP joint (7.6 degrees for hallux rigidus versus approximately 10 degrees for the current study). Similarly, Davis did not evaluate toe wedge ROM, only point by point time series comparisons [13].

Our results also confirm that coupling extends to the kinetics of the foot. During the locked brace conditions, the energy generated by the midtarsal joint and absorbed by the MTP joint were both reduced compared to unconstrained conditions. These results are congruent with previous research that showed changes in midtarsal kinetics secondary to changes in MTP alterations due to task variations [5, 15]. Specifically, when foot strike angle was modified [5] or walking speed was changed [15], kinetic changes were observed at the proximal foot joints simultaneous to changes at the MTP joint.

**Fig. 3:**
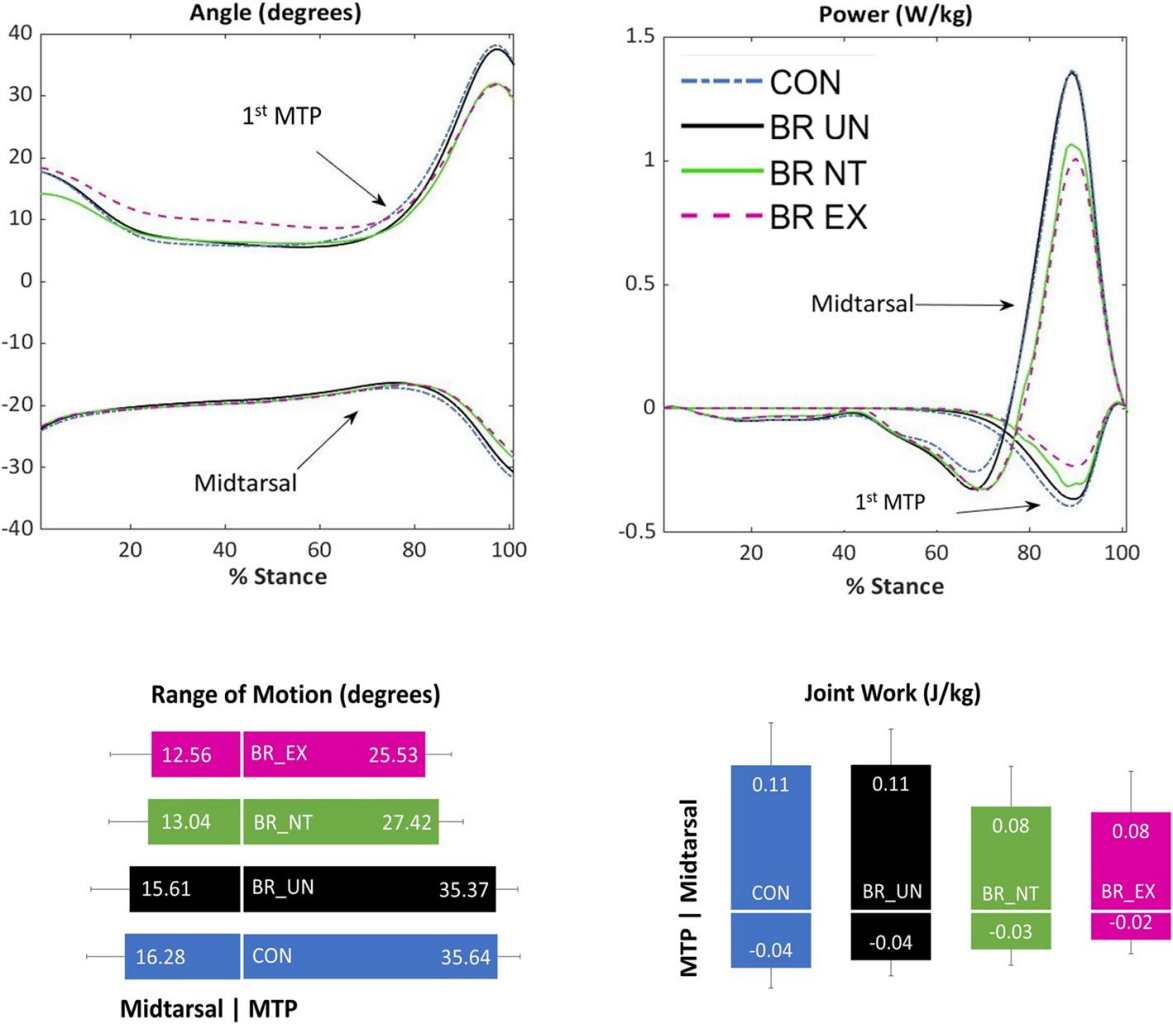
Midtarsal and MTP angle and power curves during stance phase (top row). Range of Motion (ROM; left bottom) and joint work (right bottom) values are presented as bar graphs

The magnitude and timing of the kinetic and kinematic coupling between the MTP and midtarsal joints can be visualized when plotting the angle and power profiles of both joints together (Fig. 3). For magnitudes, when ROM and joint work metrics are plotted next to each other, proportional changes in both joints are apparent (Fig. 3). While we focused primarily on quantifying signal magnitudes, timing changes may also be notable, with MTP extension and power absorption being initiated slightly later in the locked conditions and coinciding with a later midtarsal rise and transition from negative to positive midtarsal power. These plots further emphasize that when a change occurs at the MTP joint, the midtarsal joint responds with similar timing and proportional magnitudes.

**Fig. 4:**
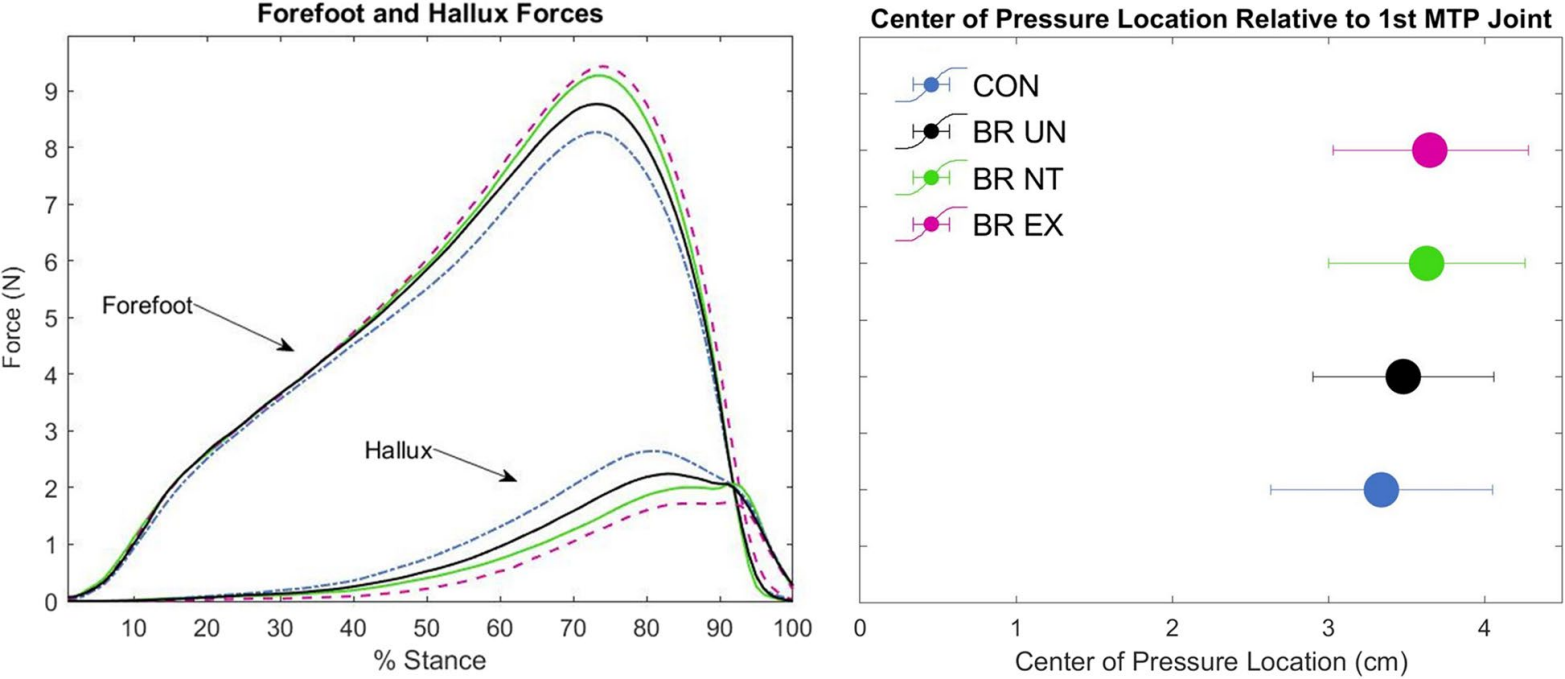
Ground reaction forces under the forefoot and hallux segments (left). Center of pressure location relative to the MTP joint center with standard deviation error bars (right)

We anticipated that a deeper look into MTP joint power components would provide additional insight into kinetic foot coupling. MTP power is the product of the joint moment and angular velocity, with the moment further subdivided into the GRF and its moment arm, represented by the COP. We noted that the hallux COP for the locked brace conditions was further anterior (Fig. 4); however, both MTP and midtarsal moments appeared to be slightly decreased in the locked conditions due to compensatory changes in segmental forces (Table 1, Fig. 2). Note that peak MTP plantarflexion moment decreased for BR_EX, but not for BR_NT due to a small late transient moment in BR_NT (Fig. 2B), which may have been caused by some noise in the FootSTEPS signals due to interactions between the walking surface and brace. To better visualize the interplay between COP and GRF for the hallux segment, we plotted the hallux COP and segmental force profiles together (Fig. 4). The locked brace conditions appeared to inhibit participants from bearing as much weight on the toe during late stance, but the location of this toe force shifted. This may have slightly inhibited forward progression by truncating the forefoot rocker. Although these subtle (and somewhat offsetting) changes in hallux COP, moment, and force were likely smaller mathematical contributions to the joint power changes in comparison with the apparent angular velocity changes, they may have at least partially caused the kinematic changes. (Note that while we didn’t measure angular velocity, substantial changes are apparent in the angle plots of Fig. 3.)

Our results provide some preliminary insights into midtarsal power generation in late stance and its associated coupling with the MTP joint; however, specific tissue contributions are still unclear. Likely midtarsal contributors include active muscle contraction or passive energy storage and return from tendons, ligaments, and fascia that cross the midfoot region. In our study, the induced changes seen at the midtarsal joint are likely primarily due to those tissues that span both the midfoot and toes, shortening this list. For instance, the arch supporting ligaments (plantar calcaneonavicular ligament and long and short plantar ligaments) and larger extrinsic muscles (tibialis posterior and fibularis longus/brevis) would primarily affect only the midtarsal joint if altered by the MTP restrictions induced in this study. Thus, main coupling contributors might be narrowed to the plantar aponeurosis and MTP flexors (flexor hallucis/digitorum longus/brevis, adductor hallucis, and quadratus plantae). While we do not rule out a role for the plantar aponeurosis and associated passive energy transfer through the windlass mechanism, previous research has suggested that its contribution to midtarsal kinematics is likely small [2, 3, 16], leaving the MTP flexor muscle–tendon units as likely primary sources of coupling.

There are two potential overlapping mechanisms for the role of the MTP flexors in MTP-midtarsal joint coupling: 1- altered muscle activation, and 2- altered joint stiffness. The brace may have inhibited MTP flexor activation or increased co-contraction, resulting in decreased muscle forces and muscle-based moments across both joints. Alternately (or in addition), the braced conditions may have altered the foot’s posture just enough to constrain the motion of both the MTP and midtarsal joints. By inhibiting forward progression, the foot may be in a disadvantageous position for generating propulsive power at the midtarsal joint; thus, similar toe flexor muscle activations would have a reduced effect on both MTP and midtarsal motion (and thus power) by increasing joint stiffness. While we did not calculate a stiffness metric, this is apparent in the similar moments but altered motion at both joints. This altered stiffness could be due to altered muscle lengths/velocities, muscle–tendon strains, or joint congruency. Future studies employing electromyography, dynamic imaging, and musculoskeletal modeling may help tease out specific mechanisms and tissue contributions.

The strong energetic coupling seen in this study suggests that interventions aimed at modulating MTP work should not ignore the midtarsal joint. As a primary example, research in footwear toe springs has suggested that reductions in MTP negative work could reflect an increase in running energetic efficiency [17], or on the flip side, may result in foot muscle atrophy [14]. While lower contraction velocities likely incur less energy cost [18], a reduction in MTP negative work does not necessarily imply a reduction in eccentric contractile velocity, as both joints crossed by these biarticular muscles are affected. A previous ultrasound study of the flexor digitorum brevis during controlled arch loading showed isometric muscle fascicles with tendon stretch making up the majority of the length changes [19]. A similar phenomenon could occur during toe extension, and it is unclear whether the brace actually changed the muscle–tendon lengths or contractile velocities. In addition, we also noted some signs that the reduced midtarsal positive work could be energetically detrimental. While not measured, there was a likely (and consequential) increase in midtarsal negative work directly preceding the reduced positive work (Figs. 2 and 3). If some of this negative work represents strain energy storage that will subsequently be returned, the toe brace resulted in a more damper-like midtarsal joint, with a higher ratio of negative to positive work [20]. The apparent increase in negative work looked to be primarily a result of a prolonged negative power phase and delayed transition to positive power. This timing change could also be detrimental from an energy storage and return standpoint, but this would need further study to confirm. Finally, the brace may have also induced subtle compensations that were not fully picked up in our analysis. While there were no differences in net knee and hip joint work, a potential reduction in total foot–ankle net positive work could be compensated for through subtle subject-specific compensations spread across multiple joints, which may not be captured in our group analysis.

Our methods had a few additional limitations that should be mentioned. As mentioned, our locked brace conditions did not fully lock the MTP joint, in either neutral or extended positions; instead, wearing the brace resulted only in slightly less peak motion for the MTP joint (~ 7 degrees). However, the decrease in peak MTP motion resulted in less midtarsal ROM (1.3 degrees) and provided the necessary stimulus to probe distal foot coupling. A fully locked brace would likely have been too extreme, resulting in large compensations elsewhere. It is also possible that the straps used to secure the brace to the foot, that wrap around and under the midfoot, could have altered pressure slightly. For this reason, we used both a no brace (CON) and unlocked brace conditions (BR_UN). Comparing the two, the brace appeared to constrain the foot slightly, with most mean curves slightly offset in the direction of the two constrained conditions. Most of these were not significant, however, and the four conditions served to provide a progression from most to least constrained, confirming the directionality and proportionality of the changes. Additionally, we only analyzed coupling within the sagittal plane. Axes within the foot are not orthogonal and investigation into other planes may offer additional insight into foot coupling [21]. For instance, Stevens et al. (2022) noted an increase in frontal plane midfoot motion during hallux rigidus [12], and it is probable that similar alterations in non-sagittal planes may have occurred in our study due to our locked brace condition. Furthermore, we should note that while we relied on descriptive analysis to define joint coupling, other more quantitative methods are available (e.g. vector coding). We felt that waveform visualization was more informative when looking at coupling mechanistically, but it may not provide as good a quantitative comparison to other studies. Lastly, there are inherent errors associated with both force measurement and biomechanical modeling. While our direct measurement approach should increase segmental force accuracy compared with prior assumption based methods [8], the technology and methodology are fairly new and may contain unanticipated errors. For modeling, joint centers and rotation axes were approximated based off markers located on the skin surface. However, previous validations of this method have shown it to be reliable with consistent marker placement [22]. The same researcher placed all markers for all subjects for this study to decrease variability.

## Conclusions

Overall, our results showed that the MTP and midtarsal joints are strongly coupled during walking, both kinematically and kinetically. This was evidenced by the consistent and proportional changes in magnitude and timing noted at both joints. We also demonstrated that foot energetics can be modulated by altering MTP motion, but the effects should not be viewed in isolation, as energy demands changed at both joints. In particular, the increase in midtarsal negative work, delayed transition to positive work, and reduced positive work need more study to fully understand their implications. This understanding is critical to our ability to enhance locomotor energetics through rehabilitation, footwear, prostheses, or other assistive devices.

## Supplementary Information

**Additional file 1.** Angle and moment waveforms for the knee and hip.

## Data Availability

The datasets used and/or analyzed during the current study are available from the corresponding author on reasonable request.

## Abbreviations

MTP: Metatarsophalangeal joint
MLA: Medial longitudinal arch
COP: Center of pressure
CON: Control
BR_UN: Unlocked brace
BR_NT: Neutral brace
BR_EX: Extended brace
GRF: Ground reaction force
ROM: Range of motion
ANOVA: Analysis of Variance
