# Supplementary figures and images for: Kinetic coupling in distal foot joints during walking
Source: J Foot Ankle Res. 2023 Jul 25;16:44. doi: 10.1186/s13047-023-00643-x (PMC10367363; doi:10.1186/s13047-023-00643-x)

## Knee

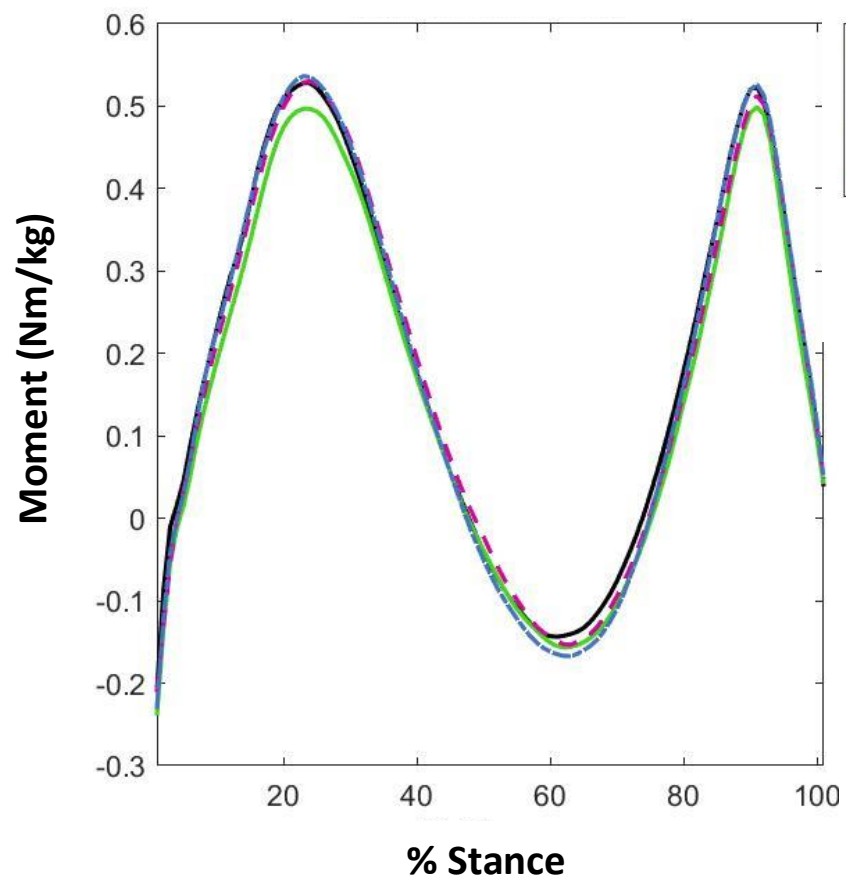

## Hip

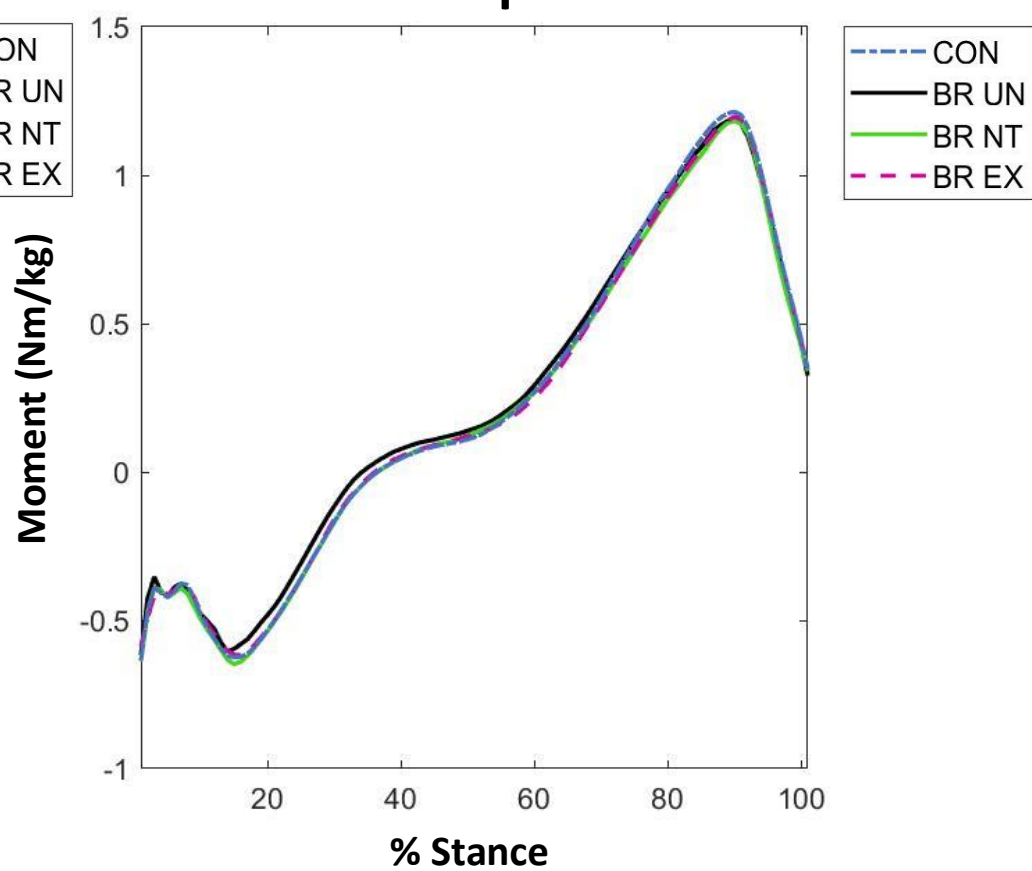

### Knee

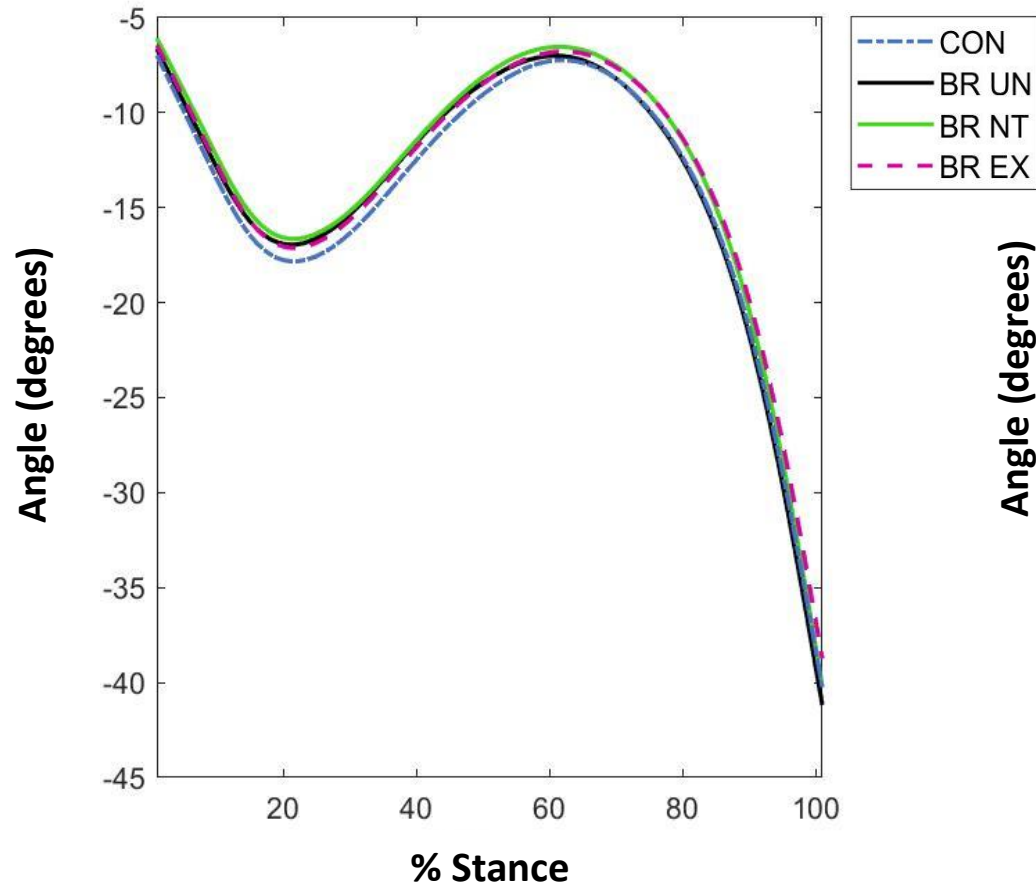

### Hip

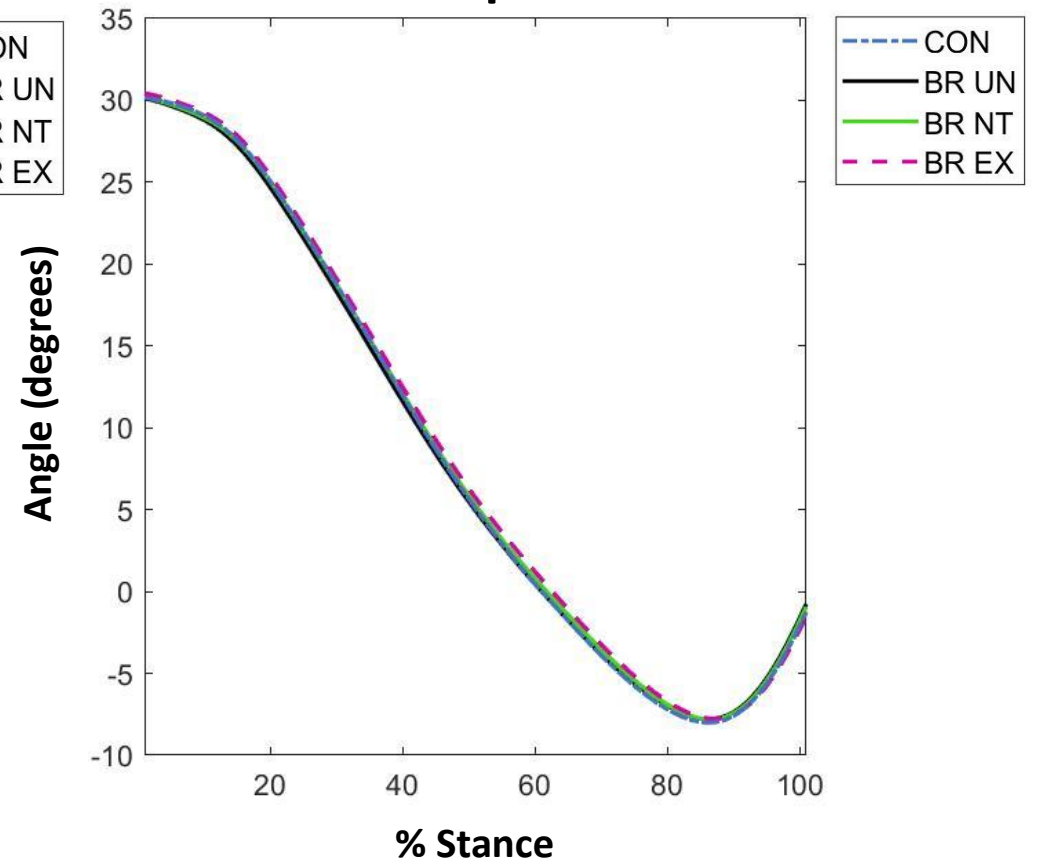

Supplement: Supplementary file 1 — Additional file 1. Angle and moment waveforms for the knee and hip. [file 13047_2023_643_MOESM1_ESM.pdf]
